# sUPRa is a dual-color reporter for unbiased quantification of the unfolded protein response with cellular resolution

**DOI:** 10.1038/s41598-024-65611-2

**Published:** 2024-07-01

**Authors:** Atreyi Chakrabarty, Sarah E. Newey, Maisha M. Promi, Belinda K. Agbetiameh, Daniella Munro, Paul J. N. Brodersen, Gemma Gothard, Kashif Mahfooz, Jose P. Mengual, Vladyslav V. Vyazovskiy, Colin J. Akerman

**Affiliations:** 1 Department of Pharmacology, University of Oxford, University of Oxford, Mansfield Road, Oxford, OX1 3QT UK; 2 Department of Physiology, Anatomy and Genetics, University of Oxford, Sherrington Building, Sherrington Road, Oxford, OX1 3PT UK

**Keywords:** Cellular imaging, Single-cell imaging, Cellular neuroscience, Sleep, Sleep deprivation

## Abstract

The unfolded protein response (UPR) maintains proteostasis upon endoplasmic reticulum (ER) stress, and is initiated by a range of physiological and pathological processes. While there have been advances in developing fluorescent reporters for monitoring individual signaling pathways of the UPR, this approach may not capture a cell’s overall UPR activity. Here we describe a novel sensor of UPR activity, sUPRa, which is designed to report the global UPR. sUPRa displays excellent response characteristics, outperforms reporters of individual UPR pathways in terms of sensitivity and kinetics, and responds to a range of different ER stress stimuli. Furthermore, sUPRa’s dual promoter and fluorescent protein design ensures that both UPR-active and inactive cells are detected, and controls for reporter copy number. Using sUPRa, we reveal UPR activation in layer 2/3 pyramidal neurons of mouse cerebral cortex following a period of sleep deprivation. sUPRa affords new opportunities for quantifying physiological UPR activity with cellular resolution.

## Introduction

Up to one-third of all proteins in a eukaryotic cell are processed in the endoplasmic reticulum (ER), where transmembrane and secretory proteins are folded and assembled^1^. The ER lumen is a tightly regulated, oxidizing environment, with high concentrations of calcium and ATP, and contains a set of molecular chaperones and enzymes that aid in the folding of proteins into their native state^2^. ER quality control systems are crucial for ensuring that misfolded proteins are retained in the ER lumen, while only proteins in their native conformation reach their destination. Endogenous or exogenous challenges to the cellular environment, such as an increase in protein synthesis or a perturbation of calcium concentrations, can lead to an accumulation of misfolded or unfolded proteins in the ER lumen, resulting in dysregulation of ER homeostasis and the onset of ER stress^1,3^. Under conditions of ER stress, an orchestrated set of signaling pathways, known as the unfolded protein response (UPR), serves as an adaptive mechanism to restore ER homeostasis. If ER homeostasis cannot be restored, the UPR can trigger apoptosis^4,5^. Detecting, monitoring and quantifying UPR activity is a key challenge for biologists studying the effects of ER stress in various physiological and disease states, and therefore there is a need for highly sensitive UPR reporters for use in vitro and in vivo.

The restorative UPR is of particular importance in the nervous system. Due to the lack of neuronal regeneration, the role of these adaptive signaling pathways in maintaining proteostasis and cell viability is essential. Failure in proteostasis and the onset of UPR-mediated apoptosis have been implicated in neurodegenerative disorders, such as Alzheimer’s disease^6,7^. Beyond pathological conditions, there is growing appreciation of how a neuron’s normal physiological processes influence ER proteostasis^8–10^. The cycling of synaptic vesicles, receptor-mediated signaling, ion transport, and physical stability of the synapse, all rely upon ER proteostasis^11^. Furthermore, synthesis of new synaptic proteins can vary as a function of an organism’s sleep–wake history^12,13^. In the brain, upregulation of genes downstream of UPR activation has been associated with wakefulness and sleep deprivation^14–20^. In turn, manipulating the UPR results in profound effects on sleep duration and intensity across multiple species^21–23^. Growing interest in the role of the UPR in physiological processes, such as sleep–wake regulation, emphasizes the challenge of detecting subtle UPR activation during normal cellular physiology compared to more robust signals in disease conditions^8,10^. Consequently, a major obstacle in studying the physiological UPR is the lack of highly sensitive UPR reporter tools.

Most recent efforts have focused on developing genetic reporters for specific UPR pathways (see Table S1 for a non-exhaustive list of mammalian UPR reporters)^24–28^. While effective for detecting the activation of a single signaling pathway, these tools may have limited overall sensitivity in capturing the global state of UPR activity within a cell. The UPR comprises three signaling pathways, or ‘arms’, transduced by three ER transmembrane sensor proteins: inositol-requiring protein-1 (IRE1), activating transcription factor-6 (ATF6) and protein kinase RNA like ER kinase (PERK)^1^. In unstressed conditions, these three sensor proteins are bound to the major molecular chaperone in the ER, binding immunoglobulin protein (BiP, also known as Grp78), maintaining them in an inactive state. At the onset of ER stress, BiP dissociates from the sensor proteins to assist in the folding of accumulated proteins in the ER lumen^29,30^, resulting in the activation of discrete downstream signaling pathways^31^. Activation of the individual arms of the UPR occurs with different dynamics and results in distinct signaling outcomes depending on the intensity and duration of ER stress. Notably, a common feature of all three arms of the UPR is the transcriptional upregulation of BiP via a number of transcription factors. BiP upregulation represents a major restorative response downstream of all three UPR arms, and its upregulation is commonly detected as a marker of the adaptive UPR, which is crucial under conditions of physiological ER stress^18,32^.

The three arms of the UPR and their convergence on BiP transcriptional upregulation can be summarized as follows. The IRE1 arm enhances protein folding through an increase in ER chaperone production and ER-associated degradation (ERAD) of accumulated unfolded proteins^1^. IRE1’s endoribonuclease activity mediates the unconventional splicing of an intron from the mRNA of the transcription factor, X-box binding protein-1 (XBP1)^33^. Spliced XBP1 (sXBP1) then transcriptionally activates a number of UPR target genes, including BiP, by binding to ER stress response element (ERSE) sequences in the target promoters^34,35^. Upon BiP dissociation, the ATF6 sensor undergoes proteolytic cleavage, resulting in an active transcription factor^36^. The active ATF6 fragment also results in transcriptional upregulation of BiP alongside other target genes by binding to ERSE promoter sequences, often in concert with sXBP1, to restore proteostasis^35,37^. Activation of the PERK arm causes a transient attenuation of protein translation and restricts entry of newly synthesized proteins into the ER to decrease the ER's protein folding load^38^. Furthermore, PERK upregulates the transcription and translation of UPR effectors that help restore proteostasis^39,40^, including the activating transcription factor-4 (ATF4), which in turn upregulates BiP transcription via the ATF/CRE binding sequence in the BiP promoter^41^.

In an attempt to capture the global UPR and generate a highly sensitive UPR reporter, we introduce a sensor of UPR activity (‘sUPRa’), which we make available to the field. This novel transcriptional reporter utilizes a short region of the mouse BiP promoter where the three arms of the activated UPR can in principle converge, to drive the transcription of the fast-maturing fluorescent protein, mNeonGreen^42,43^. This approach builds upon earlier work characterizing the transcriptional regulation, function and UPR activation of the BiP gene^32,41,44^. Incorporation of a second expression cassette that includes an UPR-independent promoter driving expression of a red fluorescent protein ensures that cells are sampled regardless of the degree of UPR activation, which is particularly important for low UPR levels under physiological conditions. sUPRa achieves a high signal-to-noise ratio, exhibits low baseline expression and superior ON–OFF dynamics, and significantly outperforms single pathway reporters of the UPR. We demonstrate that sUPRa is capable of detecting UPR activation by a range of stimuli in vitro and in vivo. Furthermore, we demonstrate sUPRa’s utility as an effective reporter of physiological UPR, as it reveals UPR activation in mouse cortical layer 2/3 pyramidal neurons following sleep deprivation.

## Results

### sUPRa: a dual color high-performance fluorescent reporter for quantifying UPR activity

**Figure 1:**
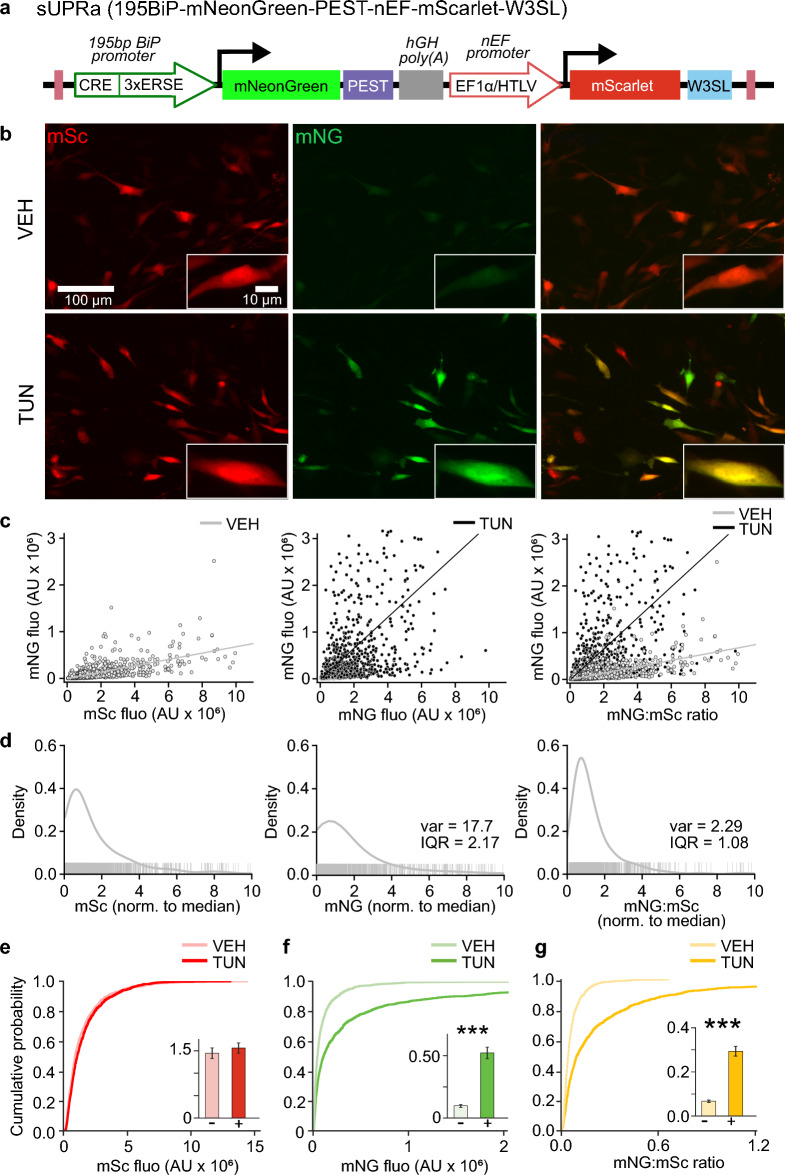
sUPRa, a dual color fluorescent reporter designed to detect UPR activation in an unbiased manner. (**A**) sUPRa encodes a bright and rapidly maturing green fluorescent protein, mNeonGreen (mNG), under the control of a fragment of the BiP promoter (bases − 195 to − 9 of the mouse gene relative to the TSS, known as 195BiP), and destabilized with a PEST sequence. In addition, sUPRa also encodes a constitutively expressed red fluorescent protein, mScarlet (mSc), under the control of an Ef1α-based promoter. mSc expression is maximized by incorporating the combined WPRE/SV40 early/late polyadenylation signal sequence, W3SL. (**B**) NIH3T3 mouse fibroblasts were transfected with sUPRa and treated with either 1:1000 DMSO VEH or 0.5 µg/ml TUN—an inducer of ER stress and the UPR—for 20 h. Images show representative fields of view of cells, with an inset of a single cell. (**C**) Scatter plots show relationship between mSc and mNG fluorescence signals for individual cells. mNG was positively correlated with mSc in both the VEH (left; n = 1236 cells from three experiments, r = 0.67, p < 0.001, Pearson’s r) and TUN (middle; n = 1214 cells from 3 experiments, r = 0.51, p < 0.001, Pearson’s r) conditions, consistent with mSc providing a readout of reporter copy number. The slope ratio of mNG to mSc fluorescence was 0.07 with VEH treatment and 0.33 with TUN treatment. (**D**) Probability density plots of mSc (left), mNG (middle), and the ratio of mNG:mSc (right) for VEH cells. Each plot is normalized to the distribution median and the vertical dashes indicate individual cells. The ratio measure accounts for variability in construct expression levels across cells, resulting in a tighter distribution with lower variance and IQR compared to the mNG distribution (n = 1236 cells from three experiments; p < 0.001, Wilcoxon signed rank test). (**E**) Cumulative distribution of mSc fluorescence in all cells, bar plots (inset) show mean ± SEM. mSc fluorescence was not altered with 0.5 µg/ml TUN treatment relative to VEH (n = 27 FOVs from three experiments; p = 0.48, t test). (**F**) Cumulative distribution and bar plot showing that mNG fluorescence is increased more than 400% following TUN treatment (n = 27 FOVs from three experiments; p < 0.001, Mann–Whitney U test). (**G**) Similarly, mean mNG:mSc ratio increased more than 400% with TUN relative to VEH (n = 27 FOVs, from three experiments; p < 0.001, Mann–Whitney U test).

Transcriptional upregulation of BiP is a common feature of UPR signaling. The key BiP promoter elements that bind to UPR-activated transcription factors are within the first 195 base pairs (bp) upstream of the transcriptional start site (TSS)^41^, including ERSE sequences, which bind sXBP1^35^ and cleaved ATF6^45^, and an ATF/CRE sequence, which binds ATF4^41^ (Fig. 1A). Expanding on previous rat BiP promoter constructs used to study ER stress-induced BiP expression^32,41,46^, we have developed a novel mouse BiP promoter-based fluorescent reporter of UPR activity, which we call ‘sUPRa’ (see “Methods”).

The BiP promoter fragment in sUPRa encompasses bases − 195 to − 9 of the mouse gene relative to the TSS^32^. This sequence drives the expression of a rapidly maturing and brighter variant of green fluorescent protein, mNeonGreen (mNG)^42,43^ (Fig. 1A). mNG is fused in-frame with the proline-glutamate-serine-threonine-rich (PEST) sequence of mouse ornithine decarboxylase (ODC). This sequence targets the fluorescent protein to the proteasome and allows for rapid turnover of mNG, providing a good temporal readout of UPR activation and deactivation in live cells^47,48^. To provide a UPR-independent signal, a second expression cassette is incorporated into sUPRa and includes the fast-maturing fluorescent protein, mScarlet (mSc), driven by the constitutively active short EF1α (nEF) promoter^49^.

Treatment with pharmacological inducers of ER stress such as Tunicamycin (TUN)—an inhibitor of N-linked glycosylation that disrupts protein folding in the ER^50^—have been widely used to elicit and study the UPR in cells^51^. Therefore, to assess sUPRa’s response to UPR induction, NIH3T3 mouse fibroblast cells were transfected with sUPRa and treated with either 0.5 µg/ml TUN or 1:1000 DMSO vehicle (VEH) for 20 h (Fig. 1B). Cells were imaged and sUPRa’s mSc signal was used for unbiased cell identification irrespective of UPR status. The mNG, mSc fluorescence, and their ratio (i.e. mNG:mSc) were then quantified for each cell. mNG levels correlated positively with mSc levels across conditions, supporting the notion that mSc offers a form of normalization for differences in reporter copy number across cells (Fig. 1C). Indeed, for cells under baseline UPR conditions, the mNG:mSc ratio values exhibited lower variance than the mNG values (Fig. 1D). The mSc signal therefore serves as an effective normalization signal, accounting for cell-to-cell variability in mNG fluorescence attributed to differences in sUPRa expression levels. sUPRa’s mSc fluorescence did not differ between VEH (1.45 ± 0.12 AU) and TUN (1.56 ± 0.11 AU) treatments (Fig. 1E). In contrast, mean mNG fluorescence showed a robust increase with TUN (0.52 ± 0.05 AU), compared to VEH (0.09 ± 0.01 AU; Fig. 1F). Following normalization to the mSc signal, the ratio of mNG:mSc was found to increase by over 400% following TUN (0.29 ± 0.02), compared to VEH (0.07 ± 0.005; Fig. 1G).

**Figure 2:**
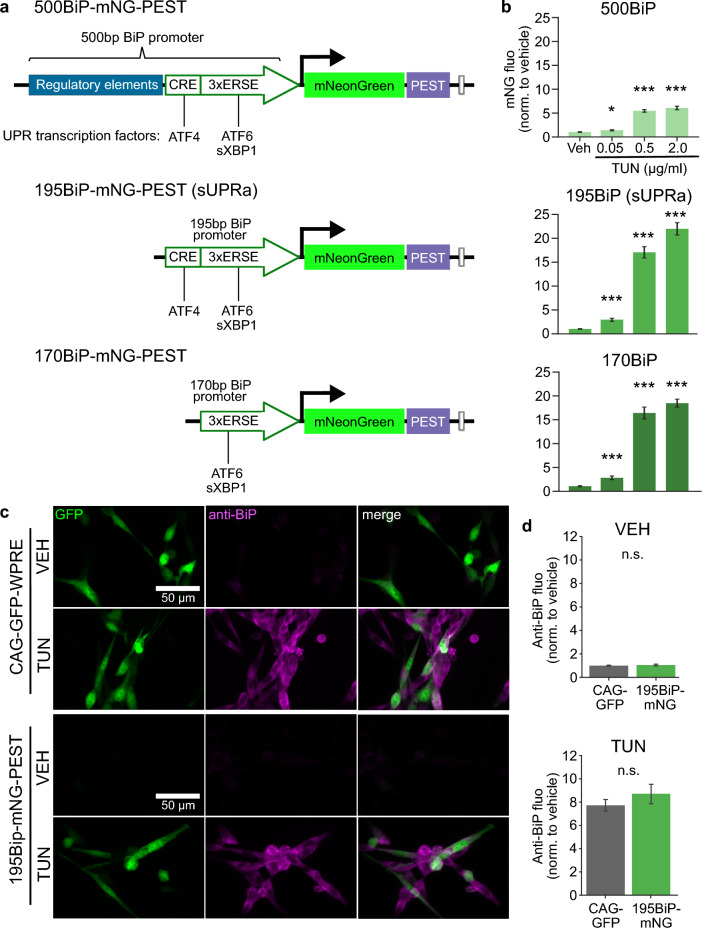
Validating sUPRa’s design and ability to capture the UPR. (**A**) Schematic diagrams of reporter constructs driving expression of mNG-PEST using 500 bp (500BiP, top), 195 bp (195BiP, middle) or 170 bp (170BiP, bottom) regions of the BiP promoter. (**B**) Mean mNG fluorescence intensity relative to vehicle from cells expressing the respective BiP promoter reporters and treated with VEH or different doses of TUN (0.05, 0.5, or 2 µg/ml) for 20 h. All reporters responded to the three doses of TUN (n = 24 FOVs from three experiments; p < 0.001, Dunn’s multiple comparisons tests comparing to vehicle, following significant Kruskal Wallis tests). Only the 195BiP-mNG-PEST reporter showed a significant increase in response between 0.5 µg/ml and 2 µg/ml TUN treatment (Fig. S1). (**C**) To test whether 195BiP-mNG-PEST interferes with endogenous UPR activation, cells expressing either 195BiP-mNG-PEST or a control construct (CAG-GFP) plasmid were treated with VEH or 2 µg/ml TUN for 20 h, after which they were immunolabelled with an anti-KDEL antibody to detect endogenous BiP protein expression. (**D**) 195BiP-mNG-PEST was no different to control in terms of baseline endogenous BiP protein, or the induction of endogenous BiP protein following TUN treatment (n = 16–24 FOVs; VEH p = 0.50, TUN p = 0.0748, t test).

The BiP promoter fragment incorporated into sUPRa was selected to maximize sensitivity to UPR induction, with low baseline expression and high signal-to-noise ratio. To establish this, we compared our novel 195 bp promoter fragment with a longer 500 bp region of the mouse BiP promoter (bases − 500 to − 9 of the mouse gene relative to the TSS), and a shorter 170 bp promoter fragment (bases − 170 to − 9) in its ability to drive mNG expression in transfected NIH cell populations (Fig. 2A). These 500 bp and 170 bp BiP promoter fragments are similar to rat sequences described in previous studies^32,44^. Cells that had been transfected with each of these constructs and treated with TUN (0.05, 0.5, 2 µg/ml), showed an increase in mNG fluorescence relative to VEH (Fig. 2B). The 500BiP promoter showed the lowest fold-induction across all doses of TUN, due to high baseline mNG fluorescence with VEH treatment (Fig. 2B, top). Meanwhile, the responses of the 170BiP and 195BiP promoters showed improved signal-to-noise due to lower baseline mNG fluorescence. While the 170BiP promoter response plateaued for the 0.5 µg/ml and 2 µg/ml TUN doses, the 195BiP promoter captured a significant increase in reporter signal between the two highest doses (Fig. 2B, Fig. S1). Thus, the 195BiP promoter offered the greatest dynamic range for UPR detection and a low baseline mNG expression, justifying its selection as the BiP promoter for sUPRa.

It was important to confirm that the 195BiP promoter fragment in sUPRa did not interfere with endogenous UPR activation mechanisms, particularly BiP protein expression. To assess this, NIH3T3 cells expressing either 195BiP-mNG-PEST or a control CAG-GFP plasmid were treated with either TUN or VEH, and the UPR was quantified using immunofluorescence detection of endogenous BiP protein (Fig. 2C). As expected, BiP immunofluorescence was upregulated with TUN treatment relative to VEH, and did not differ between cells expressing sUPRa or the control construct (Fig. 2D). Therefore, expression of sUPRa does not appear to alter endogenous UPR activation processes. Finally, to optimize sUPRa’s performance, we established that a sequential arrangement of the two expression cassettes (195BiP promoter driving mNG expression followed by the nEF promoter driving mSc) generated the brightest mNG fluorescence signal following TUN treatment (Fig. S2).

### sUPRa is highly sensitive and reflects the level of UPR activation

In order to benchmark sUPRa’s ability to detect UPR activation, we compared sUPRa’s response sensitivity to previously established fluorescent reporters of single UPR arms. One such fluorescent reporter is pCax-F–XBP1∆DBD-Venus (XBP1-Venus), which is specific for reporting activation of the IRE1 arm^25^. This reporter encodes Venus, a variant of GFP, fused to the XBP1 sequence, and relies on the unconventional splicing of an intron in the XBP1 mRNA by activated IRE1 under ER stress conditions. The splicing event results in a frameshift in the mRNA, which no longer encodes a stop codon between XBP1 and Venus, allowing translation of the XBP1-Venus fusion protein. Another more recently developed construct, SPOTlight, is a ratiometric fluorescent reporter for the PERK arm^26^. SPOTlight relies on the differential usage of open reading frames (ORFs) for translation regulation during ER stress and PERK-mediated phosphorylation of eIF2α. The sequence encoding the red fluorescent protein, TdTomato, was inserted into the ATF4 ORF, which is translated under conditions of high p-eIF2α. In contrast, enhanced GFP (EGFP) was inserted into an upstream ORF, which is translated under conditions of low p-eIF2α. Therefore, SPOTlight produces a scale-like readout of eIF2α phosphorylation downstream of PERK activation.

**Figure 3:**
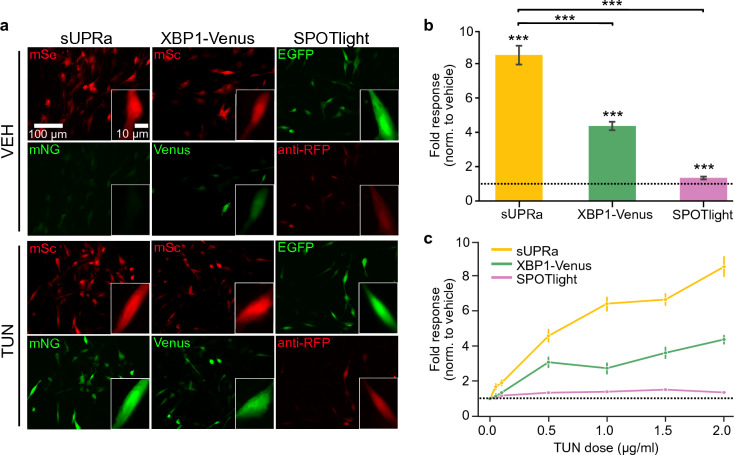
sUPRa exhibits dose-related responses and greater sensitivity than single-arm UPR reporters. (**A**) NIH3T3 cells expressing either sUPRa, XBP1-Venus or SPOTlight, were treated with VEH or 2 µg/ml TUN for 20 h. SPOTlight-expressing cells were immunolabelled with an anti-RFP antibody to amplify the RFP signal. Images show representative fields of view of cells with an inset of a single cell. (**B**) Plot shows each reporter’s fold response to 2 µg/ml TUN, normalized to VEH. All three reporters showed a response to TUN (n = 16–24 FOVs from at least two experiments; p < 0.001, Mann–Whitney U tests), with sUPRa showing a greater response than either XBP1-Venus or SPOTlight (n = 16–24 FOVs from at least two experiments; p < 0.001, Mann–Whitney U tests). sUPRa response was measured as the ratio of mNG to mSc fluorescence; XBP1-Venus response was the level of Venus fluorescence; SPOTlight response was the ratio of anti-RFP to EGFP fluorescence. (**C**) Responses induced by a series of different TUN doses (0, 0.05, 0.1, 0.5, 1 and 2 µg/ml), relative to VEH for each reporter (data represented as mean ± SEM of n = 16–24 FOVs from at least two experiments). All sensors show a dose-dependent response to the range of TUN doses (0, 0.05, 0.1, 0.5, 1 and 2 µg/ml) (p < 0.001, Kruskal–Wallis test for dose effect). sUPRa exhibited a greater response than XBP1-Venus and SPOTlight at all TUN doses (p < 0.05, Mann Whitney U tests with Bonferroni correction).

Cells were transfected with either sUPRa, XBP1-Venus or SPOTlight. Since XBP1-Venus provides only a green fluorescence readout, these cells were co-transfected with equal amounts of a nEF-mScarlet-W3SL plasmid to allow automated cell detection on an UPR-independent channel. Cells were then treated for 20 h with TUN (0.05, 0.1, 0.5, 1.0, 1.5, 2.0 µg/ml) or DMSO vehicle. Subsequently, the fluorescence response of each reporter was quantified. For sUPRa, the response was defined as the mNG:mSc fluorescence ratio. For XBP1-Venus, the response was defined as the relative change in Venus fluorescence. Consistent with previous work, the TdTomato expression from SPOTlight was low and required amplification with an anti-RFP antibody^26^, which enabled the SPOTlight response to be defined as the TdTomato:EGFP fluorescence ratio (Fig. 3A). All three reporters exhibited a significant response to TUN, but sUPRa exhibited the greatest response overall, which was more than 200% greater than the best single-arm reporter (Fig. 3B and Fig. S3) Furthermore, sUPRa was the only reporter to respond to the lowest dose of TUN, 0.05 µg/ml, indicative of its potential to report physiological levels of UPR^52^ (Fig. 3C). sUPRa displayed the greatest fold-response across all doses of TUN compared to XBP1-Venus and SPOTlight, thereby demonstrating sUPRa's superior dynamic range and signal-to-noise ratio compared to these single-arm UPR reporters.

### sUPRa can track UPR dynamics

**Figure 4:**
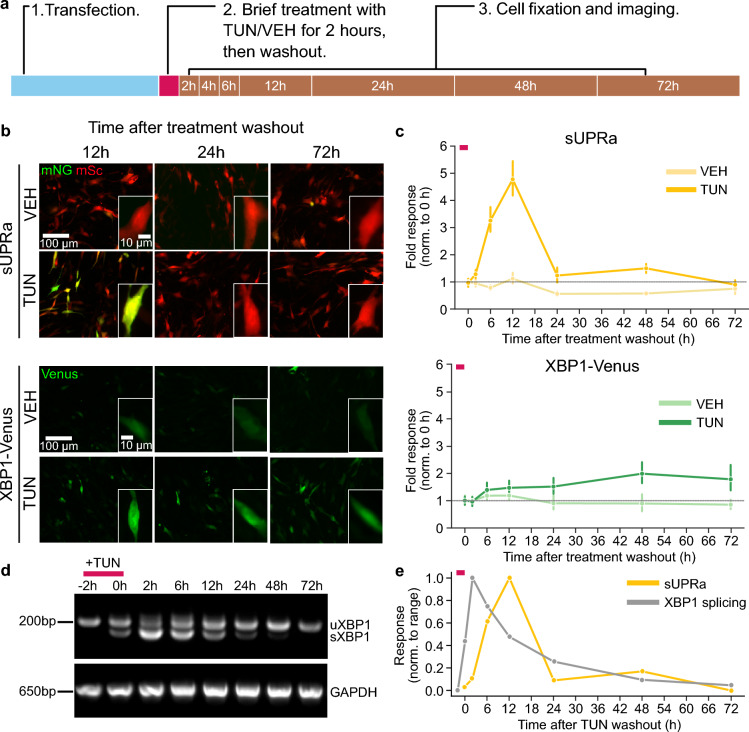
sUPRa tracks UPR dynamics in response to transient ER stress induction. (**A**) NIH3T3 cells expressing either sUPRa or XBP1-Venus were treated with VEH or 2 µg/ml TUN for 2 h. The treatment was then washed out and cells were fixed and imaged at different timepoints. (**B**) Images show representative fields of view of cells, with an inset of a single cell expressing sUPRa (top) or XBP1-Venus (bottom) at 12, 24 and 72 h after treatment washout. (**C**) Response dynamics of sUPRa (top) and XBP1-Venus (bottom) relative to treatment washout (data represented as mean ± SEM from n = 24 FOVs from three experiments per timepoint). Red dash marks the duration of treatment with TUN/VEH. sUPRa response to TUN peaked at 12 h and returned to baseline by 24 h after washout (p < 0.001, 2-way ANOVA for timepoint and treatment effect; p < 0.001 comparing 0 and 12 h, and p = 0.9 comparing 0 and 24 h, post hoc Tukey HSD). XBP1-Venus response remained high 72 h after treatment washout (p < 0.001, 2-way ANOVA for timepoint and treatment effect; p = 0.0135 comparing 0 and 72 h, post hoc Tukey HSD). (**D**) Agarose gel showing endogenous XBP1 transcript splicing in response to a 2-h TUN treatment (between -2 and 0 h), before washout and recovery. GAPDH RT-PCR is shown as a control. (**E**) Comparison of response dynamics for sUPRa and endogenous XBP1 splicing. Red dash marks the duration of treatment with TUN. XBP1 splicing data shows the proportion of spliced XBP1 (sXBP1) relative to the total XBP1 at a given time point, normalized to the range. sUPRa response, from '**C**', was also normalized to its range.

In addition to reporting UPR activation at a given time point, sUPRa was designed with ON–OFF dynamics in mind, by incorporating the rapidly maturing mNG and a protein destabilizing sequence. To illustrate this, we tested the ability of sUPRa and XBP1-Venus to report the onset and offset of UPR activation following a transient TUN treatment. Cells were exposed to VEH or 2 µg/ml TUN for a 2-h period, and then imaged 0, 2, 6, 12, 24, 48 or 72 h after treatment washout (Fig. 4A). In response to this transient UPR activation, both sUPRa and XBP1-Venus displayed a time point and treatment-dependent increase in response (Fig. 4B), confirming that both reporters are sufficiently sensitive to report the UPR elicited by the 2-h treatment.

However, sUPRa exhibited a faster and larger peak response, plus a faster recovery to baseline compared to XBP1-Venus (Fig. 4C and Fig. S4). A strong sUPRa reporter signal was observed 6 h post-treatment, with the peak response observed at 12 h (4.71 ± 0.26), and a return to baseline by 24 h (1.19 ± 0.09). Meanwhile, the XBP1-Venus fold-response was slower to peak at 48 h (1.63 ± 0.09) and remained high 72 h after treatment. Thus, sUPRa exhibits improved performance in terms of its ability to provide a readout of UPR onset and offset dynamics. To compare sUPRa’s ON–OFF dynamics with the endogenous UPR, we performed the same time-course experiment and assessed endogenous XBP1 RNA splicing using RT-PCR (Fig. 4D,E and Fig. S5). This revealed that the ON rate for maximal XBP1 splicing was slightly faster than for maximal sUPRa activation, which is consistent with the requirement for transcription and translation of the fluorescent reporter proteins to generate the sUPRa signal. Meanwhile, the OFF rates for XBP1 splicing and sUPRa exhibited similar timescales, with both returning to 25% or less of the maximal signal by 24 h after TUN washout. These data demonstrate that sUPRa’s design makes it well suited for capturing UPR dynamics.

### sUPRa responds to UPR activation by misfolded proteins and multiple chemical ER stressors

**Figure 5:**
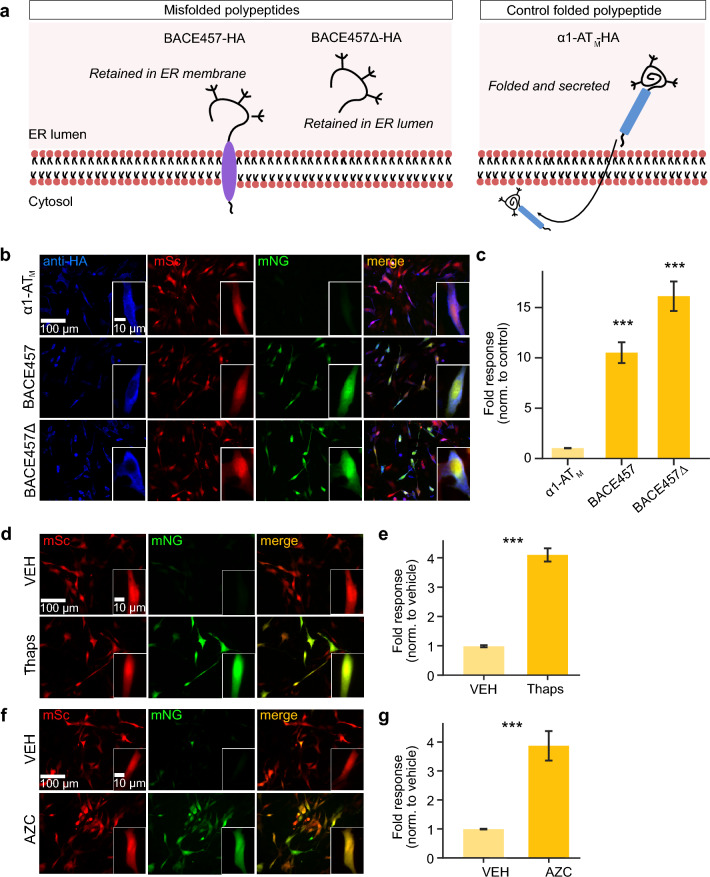
sUPRa responds to different forms of ER stress induction. (**A**) ER stress and the UPR were induced in NIH3T3 cells by the expression of mutant misfolded polypeptides (left), which are retained in the ER membrane (BACE457) or ER lumen (BACE457∆). Control cells expressed a folded polypeptide that does not accumulate in the ER (α1-AT_M_). (**B**) Images show representative fields of view of cells, inset shows a single cell co-expressing sUPRa and the polypeptides shown in ‘**A**’. An anti-HA antibody was used to detect polypeptide expression. (**C**) sUPRa shows more than a tenfold response to both forms of misfolded polypeptide compared to the control polypeptide, with the ER luminal polypeptide, BACE457∆, inducing the strongest UPR response (n = 24 FOVs from three experiments; p < 0.001, Mann Whitney U tests). (**D**) Cells expressing sUPRa were treated with either Thapsigargin (Thaps), a SERCA pump blocker, or VEH (DMSO 1:1000). (**E**) ER stress induction with Thaps produced a fourfold increase in sUPRa response relative to VEH (n = 17 FOVs from two experiments; p < 0.001, t test). (**F**) Cells expressing sUPRa were treated with either L-Azetidine carboxylic acid (AZC), a proline analog and inducer of protein misfolding, or VEH (dH_2_O). (**G**) ER stress induction with AZC produced a fourfold increase in sUPRa response relative to VEH (n = 17 FOVs from two experiments; p < 0.001, t test).

The UPR can be triggered by various factors that activate one or more of its three arms to different degrees^4,5,10^. Having established that sUPRa reports UPR activation by the N-glycosylation inhibitor, Tunicamycin, we wanted to confirm that sUPRa reports UPR activation elicited by other means. Whilst pharmacological agents have been well-established to induce the UPR indirectly by altering ER function and causing protein misfolding, they are also likely to induce pleiotropic effects^52^. An alternative drug-free approach to directly induce the UPR in a cell-targeted way is to express misfolded proteins. Such genetic tools have been well-characterized and reported to induce a physiological and specific UPR^52,53^. To test whether sUPRa reports UPR induction caused by misfolded proteins, sUPRa-expressing cells were co-transfected with either a misfolded polypeptide construct or a control folded polypeptide construct. We used two misfolded variants of pancreatic β-secretase in the form of BACE457 and its soluble variant, BACE457∆, which are retained in the ER in an immature state^53^ (Fig. 5A). The effects were compared to control cells expressing a correctly folded serine protease inhibitor, α1AT_M_, which matures in the ER and is released^54^. The polypeptides incorporate an HA-tag sequence and so expression was confirmed using a HA-tag antibody (Fig. 5B). Compared to α1AT_M_, sUPRa showed a 10.51 ± 1.13 and 16.13 ± 1.56 fold-increase with BACE457 and BACE457∆, respectively (Fig. 5C). Thus, sUPRa reports robust UPR activation induced directly by misfolded proteins.

There are a number of other well-characterized pharmacological inducers of the UPR. Thapsigargin (Thaps) is an inhibitor of the sarco/endoplasmic reticulum Ca2 + ATPase (SERCA), which elicits a UPR^3,55^. Meanwhile, l-azetidine-2-carboxylic acid (AZC) is a proline analog known to induce protein aggregation in the ER^56^. To investigate whether sUPRa responds to these different ER stress inducers, sUPRa-expressing cells were treated with either 25 nM Thaps, 0.5 mM AZC, or the appropriate VEH control for 20 h (Fig. 5D,F). Both the Thaps and AZC treated cells exhibited a ~ 400% increase in sUPRa response relative to VEH (Fig. 5E,G). These results confirm that sUPRa detects a range of ER stress-inducing stimuli, supporting its use as a general reporter of a cell’s UPR.

### sUPRa reports UPR activation in mouse cortical pyramidal neurons

**Figure 6:**
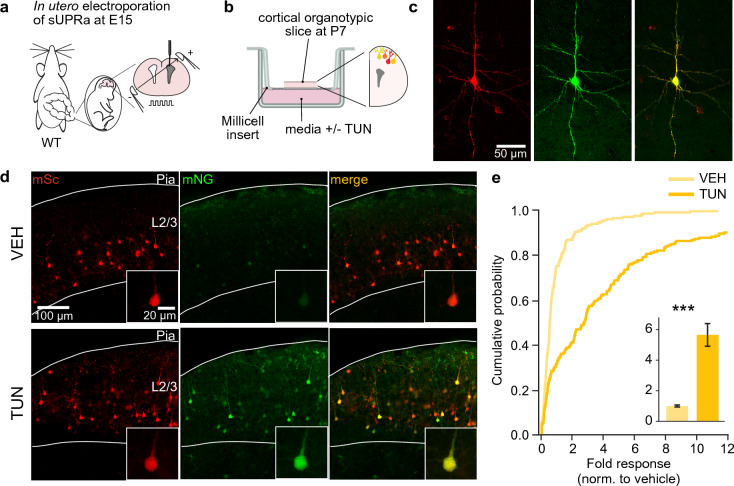
sUPRa responds to Tunicamycin-induced ER stress in mouse cortical neurons. (**A**) sUPRa expression was targeted to L2/3 pyramidal neurons in mouse cortex, by performing in utero electroporation on E15 mouse embryos. (**B**) Cortical organotypic slices were generated at P7, and treated with either 5 µg/ml TUN or VEH for 6 h and prepared for imaging 18 h later. (**C**) A L2/3 pyramidal neuron expressing sUPRa in a TUN-treated cortical organotypic slice at P7. (**D**) Representative images of sUPRa expression in L2/3 pyramidal neurons following VEH or TUN treatment. Low power image shows L2/3 in an organotypic slice, and inset shows individual pyramidal neuron. (**E**) Cumulative distribution of sUPRa’s fold response in VEH-treated cells (n = 197 cells from 3 slices) and TUN-treated cells (n = 211 cells from 3 slices). Bar plot (inset) show mean ± SEM. sUPRa’s response was ~ 600% higher in L2/3 pyramidal neurons treated with TUN (p < 0.001, Mann Whitney U test).

Given the importance of the UPR in restoring ER stress in neurons, we tested whether sUPRa is able to report UPR levels in a defined neuronal population. In utero electroporation was used to target the expression of sUPRa to excitatory pyramidal neurons in layer 2/3 (L2/3) of mouse cerebral cortex. This involved delivering sUPRa plasmid DNA to a population of dividing progenitor cells in the ventricular zone of mouse embryos at embryonic gestation day 15 (E15), when cortical L2/3 pyramidal neurons are being born^57^ (Fig. 6A). Subsequently, organotypic cortical slices were prepared and cultured from electroporated mice at postnatal day 7 (P7), providing an ex vivo model system to examine the UPR^58,59^. Slices were cultured for 5 days before being treated with either 5 μg/ml TUN or VEH for 6 h and then prepared for imaging 18 h later (Fig. 6B). sUPRa showed good expression in L2/3 pyramidal neurons (Fig. 6C) and exhibited a robust response to UPR activation with TUN (Fig. 6D). Cortical pyramidal neurons in slices treated with TUN showed ~ 600% increase in sUPRa response relative to vehicle (Fig. 6E), thereby supporting the use of the reporter in quantifying the UPR in mouse neuronal populations.

### sUPRa reveals a UPR in L2/3 cortical pyramidal neurons following sleep deprivation

**Figure 7:**
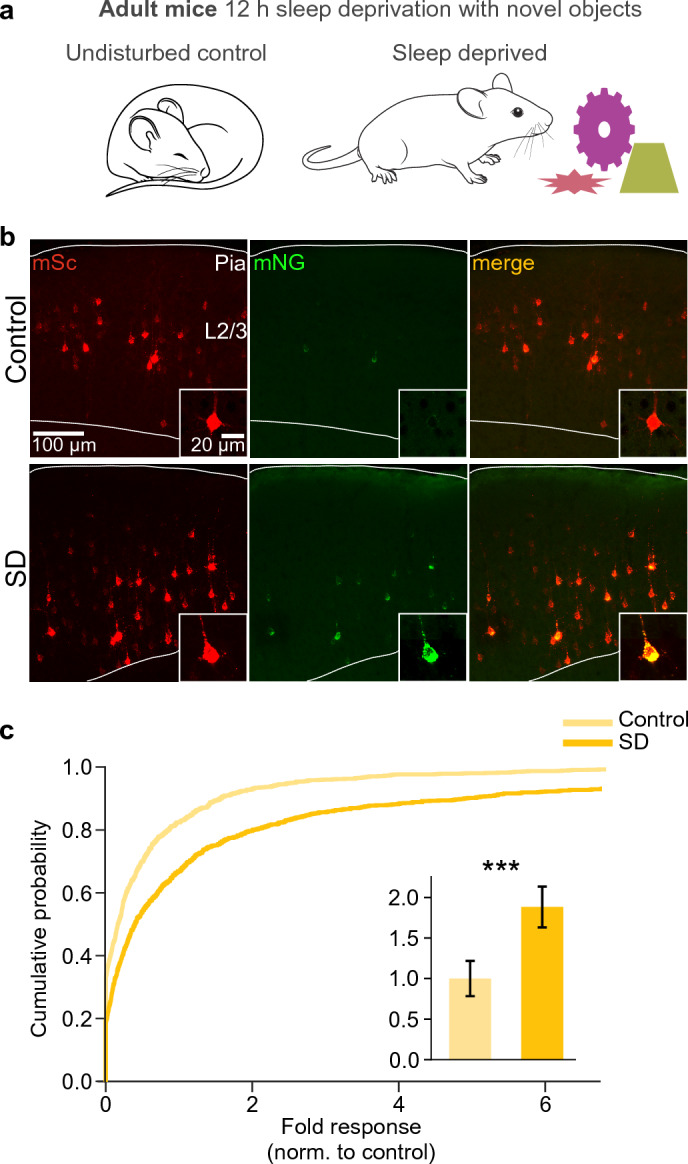
sUPRa reveals a sleep deprivation-induced UPR in L2/3 cortical pyramidal neurons. (**A**) At postnatal age 8 weeks, mice expressing sUPRa in L2/3 pyramidal neurons were either allowed to sleep under undisturbed conditions or experienced sleep deprivation (SD) for 12 h from light onset, by exposure to a series of novel objects. (**B**) Representative images of sUPRa expression in L2/3 pyramidal neurons in sleep-deprived mice or time-matched undisturbed control mice. Low power image shows L2/3 in a 50 µm thick section from somatosensory cortex, inset shows an individual L2/3 pyramidal neuron. (**C**) Cumulative distribution of sUPRa’s fold response in control mice (n = 1080 cells from 4 animals) and SD mice (n = 980 cells from 4 animals). Bar plot (inset) shows mean ± SEM. The sUPRa response in L2/3 pyramidal neurons from SD mice was increased by 88%, compared to rested controls (p < 0.001, Mann Whitney U test).

Having established sUPRa’s high sensitivity to a range of ER stress-inducing factors, we wanted to test whether sUPRa could report ER stress that is associated with in vivo physiological processes in a defined cell population. Biochemical methods have previously shown a tissue-wide increase in the UPR in mouse cerebral cortex following a period of sleep deprivation (SD)^17,18,20^. However, the cellular resolution of sUPRa offers the potential to investigate cell type-specific UPR activation following SD. To explore this potential, we examined the effects of a naturalistic SD paradigm in adult mice expressing sUPRa in L2/3 cortical pyramidal neurons (Fig. 7A). The mice were either allowed to sleep undisturbed (control group) or were kept awake by exposure to novel objects for 12 h from light onset, when they would normally be sleeping (SD group). sUPRa showed successful expression in L2/3 pyramidal neurons of 8 week-old mice that had been electroporated at E15, confirming its potential for long-term neuronal expression in vivo (Fig. 7B). Furthermore, when comparing neurons across the two conditions, we observed an 88% increase in sUPRa response in mice that had experienced SD compared to the undisturbed control mice (Fig. 7C). This demonstrates the ability of sUPRa to report physiologically relevant UPR activity in an intact in vivo system.

## Discussion

To better understand the contribution of the unfolded protein response (UPR), it is crucial to detect and quantify the UPR in individual cells under physiological and pathological conditions, in an unbiased manner. To address this, we have developed sUPRa, a dual color transcriptional reporter designed to quantify a cell’s global UPR activity. sUPRa has been demonstrated to show excellent response characteristics with a sensitive and dose-related readout of UPR activation, and exhibits effective onset and offset kinetics as the UPR initiates and dissipates. sUPRa reports the UPR elicited by different forms of ER stress, including disruptions to protein folding by inhibition of N-glycosylation or incorporation of a non-amino acid proline analog, alterations to calcium homeostasis, or the expression of ER-retained misfolded proteins. Finally, we capitalize upon the cellular-resolution imaging afforded by sUPRa to reveal that pyramidal neurons in mouse cerebral cortex exhibit an UPR following an in vivo and physiologically-relevant manipulation of sleep–wake history*.*

sUPRa incorporates a novel region of the mouse BiP promoter sequence, encompassing both ERSE sequences and a ATF/CRE binding site. BiP chaperone upregulation is one of the most important adaptive outcomes of the UPR, which is also most relevant in physiological conditions. The selection of the BiP promoter is likely to underpin sUPRa’s high sensitivity since it is a potential convergent point for capturing all three arms of the UPR. Moreover, the short BiP promoter region is devoid of accessory regulatory elements, reducing baseline expression in the absence of ER stress and dramatically improving signal-to-noise ratio. To assess sUPRa’s performance, it was benchmarked against a reporter containing a shorter fragment of the BiP promoter (170BiP-mNG-PEST) and two reporters of individual UPR arms. Compared to 170BiP-mNG-PEST, the 195BiP promoter fragment of sUPRa was able to capture a significant increase in reporter signal between the highest two doses of TUN treatment, most likely reflecting the inclusion of the ATF4 binding site in sUPRa^41^. sUPRa also showed superior response properties to a range of UPR activation states compared to a reporter of XBP1 splicing downstream of the IRE1 arm^25^ and a reporter of the PERK arm^26^. This is consistent with the idea that transcriptional activation of the BiP promoter downstream of the three UPR arms allows for a cumulative signal that is greater than any one arm alone^35^, and establishes sUPRa as a highly sensitive reporter for imaging the UPR with cellular resolution. Since sUPRa is designed to capture a cell’s global UPR and exhibits high sensitivity to different ER stress-inducing stimuli*,* the reporter’s principal strength may be in determining whether an UPR is elicited under particular conditions, after which the contribution of the individual UPR arms could be further dissected using other available tools.

A reporter should ideally match the dynamics of the endogenous process it is detecting^60^. We demonstrated that sUPRa showed a distinct ON–OFF profile in response to a transient UPR activation, which followed the profile of endogenous XBP1 splicing. This strong transient response was not evident with the XBP1-Venus reporter, which may reflect a difference in the stability of the different reporter fluorescent proteins. sUPRa was designed for optimal ON kinetics by incorporating the rapidly maturing fluorescent protein, mNeonGreen, which has a maturation speed over five-fold faster than other GFPs^42,61^. To enhance sUPRa’s OFF kinetics, a PEST degradation signal peptide was fused to mNeonGreen, reducing its half-life from approximately 20–2 h^62,63^. The increased turnover results in lower baseline expression, contributing to a better signal-to-noise ratio. Destabilized fluorescent protein reporters commonly suffer from reduced signal intensity at any given time. However, incorporation of the super bright mNG into sUPRa was confirmed to provide a strong signal in both cell culture and denser brain tissue.

As a dual promoter and dual color reporter, sUPRa was designed to ensure unbiased cell detection irrespective of UPR levels. Using a single fluorescent protein reporter is sufficient when all cells are known to express it or when all expressing cells are expected to display UPR activation to the same extent. If this is not the case however, a second UPR-independent signal is required for cell identification to avoid introducing bias towards detecting higher UPR activation levels. This is especially important under physiological conditions, as illustrated in our sleep deprivation experiment, where a high proportion of neurons exhibited no detectable UPR activation. sUPRa’s stably expressed red fluorescent protein can also account for cell-to-cell variability in reporter expression levels due to copy number, which is particularly useful for non-stable expression systems. sUPRa was engineered within an AAV backbone to enable the potential for viral-mediated delivery of the reporter in vivo. However, reports that recombinant AAV infection can itself activate all three arms of the UPR and upregulate BiP transcript levels^64^ led us to use in utero electroporation of plasmid DNA for our initial in vivo characterization. Further experiments will be required to establish whether sUPRa can be delivered using AAVs to report the UPR in vivo.

A major motivation behind developing sUPRa was to investigate the UPR under non-pathological conditions. Neurons, being unable to regenerate, are vulnerable to stresses arising from multiple physiological mechanisms, including challenges associated with neuronal activity and synaptic transmission^8,65^. To maintain normal function and viability, neurons rely on continuous prophylactic quality control mechanisms. Sleep has been proposed to facilitate essential restorative processes in the brain^66^, as evidenced by biochemical observations linking periods of sleep deprivation to a tissue-wide UPR in the brain^18,20,67,68^. Here, we leveraged the cellular resolution afforded by sUPRa to investigate whether sleep deprivation activates the UPR in a defined cell population within mouse cortex. By targeting sUPRa to layer 2/3 pyramidal neurons, it revealed a cell-type specific UPR following sleep deprivation. This proof-of-principle establishes the basis for future work that could use sUPRa to determine how distinct cell populations differ in their sleep–wake related UPR, with the potential to capitalize on sUPRa’s favorable dynamics in order to track cell-specific UPR across different behavioral states. Given its viable long-term expression, sUPRa is well-suited for monitoring the UPR in models of development, ageing, and behavioral tasks that assess cognitive processes such as learning and memory.

In summary, we present sUPRa as a resource to measure overall UPR activity in a cell-specific manner. We believe sUPRa will be an important tool for understanding ER stress and the UPR in a variety of contexts, including physiological prophylactic functions that preserve cell viability, and pathological functions in disease models and ageing.

## Methods

### Cell lines

NIH3T3 mouse embryonic fibroblast cells, derived from a mouse NIH/Swiss embryo (ECACC General Cell Collection, RRID: CVCL_0594), were maintained at 37 °C and 5% CO_2_ in Dulbecco’s modified Eagle medium (DMEM) with high glucose and pyruvate, supplemented with GlutaMAX (Gibco, Thermo Fisher Scientific) and 10% bovine serum (Gibco, Thermo Fisher Scientific) and passaged at 90% confluence.

### Constructs and cloning

sUPRa is a dual promoter construct designed to encode the green fluorescent protein, mNeonGreen, under the control of BiP/GRP78 mouse promoter fragment and a red fluorescent protein, mScarlet, under the control of a constitutively expressed nEF promoter. The BiP/GRP78 promoter fragment in sUPRa encompasses − 195 to − 9 of the mouse gene relative to the transcriptional start site^32,41^. The constructs 500BiP-mNG-PEST and 170BiP-mNG-PEST encompass promoter fragments − 500 to − 9 and − 170 to − 9 respectively. The design of these BiP/GRP78 promoter constructs was informed by the work of Amy S Lee and colleagues using rat promoter reporter constructs^41^. The sUPRa construct generated in this study is made available via Addgene (Addgene_223242; https://www.addgene.org/).

sUPRa was constructed using the vector pAAV-CAG-mNeonGreen (gift from Viviana Gradinaru; RRID: Addgene_99134) as a starting point^69^. The woodchuck hepatitis virus post-transcriptional regulatory element (WPRE) sequence was removed using a BsrGI/XhoI digest. The mouse ornithine decarboxylase (ODC) PEST sequence^47^, encompassing the carboxy-terminal 39 amino acids of ODC (NCBI Reference Sequence: NM_013614.3), was PCR amplified from an in-house mouse brain cDNA library and inserted in-frame with the C-terminus of mNeonGreen in the plasmid vector. The CAG promoter was removed using MluI/KpnI and replaced with the -195 base pair BiP/GRP78 mouse promoter fragment, which was amplified from genomic DNA purified from mouse cerebellum, to generate 195BiP-mNG-PEST. The constructs 500BiP-mNG-PEST and 170BiP-mNG-PEST were constructed in the same way using different fragments of the mouse BiP/GRP78 promoter. All PCR products for cloning were generated using Q5 High Fidelity DNA polymerase (New England Biolabs) and digests were performed using restriction enzymes from New England Biolabs. Vectors were dephosphorylated using Antarctic phosphatase (New England Biolabs), ligations were performed with DNA Ligation Kit Mighty Mix (Takara Bio) and transformations carried out using Max Efficiency Stbl2 competent cells (Thermo Fisher Scientific). All promoters and inserts were fully sequenced (Source Bioscience).

The second step for constructing sUPRa was to insert a second expression cassette encoding the constitutively expressed red fluorescent protein mScarlet into 195BiP-mNG-PEST. To this end, we constructed the vector pAAV-nEF-mScarlet-W3SL, which consists of the short, constitutively expressed nEF promoter, the sequence encoding mScarlet-I^70^ and the W3SL sequence, which is a modified and compact combined WPRE and SV40 late polyadenylation signal sequence^71^. Starting with the vector pAAV-hSyn-DIO{ChETA-mRuby2}on-W3SL (gift from Adam Kepecs; RRID: Addgene_111389)^72^, the hSyn promoter was removed using MluI/SalI and replaced with the nEF promoter, which was PCR amplified from the construct pAAV-nEF Con/Foff hChR2(H134R)-EYFP (gift from Karl Deisseroth; RRID: Addgene_55647)^49^. The insert and LoxP sites were subsequently removed using SalI/EcoRI and replaced with mScarlet-I, amplified from pCytERM-mScarlet-I-G-GECO1.2 (a gift from Dorus Gadella; RRID: Addgene_85068). To make sUPRa, the nEF-mScarlet-W3SL cassette was PCR amplified and inserted into a PmlI site in 195BiP-mNG-PEST. The orientation of insertion of the nEF-mScarlet-W3SL cassette was confirmed by restriction digest and sequencing of both expression cassettes. Reporter plasmids used alongside sUPRa were gifted by Dr Masayuki Miura (pCAX-F-XBP1∆DBD-venus)^25^ and Dr Nicole Calakos (AAV-CAG-SPOTlight-U2GCR, RRID: Addgene_164819)^26^. Recombinant polypeptide plasmids used alongside sUPRa were gifted by Dr Maurizio Molinari (pcDNA3-CMV-BACE457-HA, pcDNA3-CMV-BACE457∆-HA and pcDNA3-CMV-α1-AT_M_-HA)^52^.

### Cell culture transfection and treatment

13 mm glass coverslips were placed in 24-well plates, incubated with 0.1 mg/ml poly-d-lysine (Sigma-Aldrich) for 1 h at 37 °C, then washed and air-dried. For experiments, NIH3T3 cells seeded onto the coverslips at a density of 1 × 10^6^ cells per plate. The following day, at 60–80% confluency, the cells were transfected with the indicated plasmids using Lipofectamine 2000 (Invitrogen, Thermo Fisher Scientific), as per the manufacturer’s instructions. The media was changed after 3 h and the cells were left overnight before further treatment. Tunicamycin (Sigma-Aldrich) was prepared as a 5 mg/ml stock in dimethyl sulfoxide (DMSO, Sigma-Aldrich) and further diluted in cell culture media. Thapsigargin (Sigma-Aldrich) was prepared as a 1 mM stock in DMSO and further diluted in cell culture media. l-Azetidine carboxylic acid (Sigma-Aldrich) was prepared as a 50 mg/ml stock in dH_2_O and further diluted in cell culture media. Final concentrations of drug solutions and incubation times are indicated where relevant. Following the treatment period, cells were pre-fixed in 2% paraformaldehyde (PFA, Sigma-Aldrich) in 0.1 M PBS for 10 min, followed by fixation in 4% PFA for 10 min.

### XBP1 semi-quantitative RT-PCR

NIH-3T3 cells were seeded onto 6 well plates at 5 × 10^5^ cells per well. The next day, cells were treated with 2 µg/ml TUN for 2 h after they were washed with fresh media (time 0 h). Cells from 2 wells were harvested in RPE buffer (RNeasy kit, Qiagen) at each time point indicated and frozen at − 80 °C until all samples were collected. Samples were processed for total RNA extraction and 2 µg of total RNA was used to generate first strand cDNAs using the SuperScript III First Strand Synthesis SuperMix (Invitrogen) according to the manufacturer’s instructions. 2 µl of first strand was used in each PCR reaction and run for 25 cycles. The XBP1 primers used were: mXBP1 Fwr (GAACCAGGAGTTAAGAACACG) and mXBP1 Rev (AGGCAACAGTGTCAGAGTCC). GAPDH PCRs were performed as a control using primers hGAPDH Fwr (ATCCCATCACCATCTTCCAG) and GAPDH Rev2 (CACCACCCTGTTGCTGTAG). PCR products were resolved on a 3% agarose gel stained with GelGreen (Biotium) and photographed using a Fairphone 3 camera. XBP1 splicing was quantified using a non-saturated image of the gel and by determining the percentage of spliced XBP1 transcript as a fraction of the total XBP1 transcript for each time point using the Gel Analysis function of Image J. To compare the response dynamics of sUPRA and XBP1 splicing, the relevant data sets were normalized to their maximum response.

### Animals and ethics

All mice were bred, housed and used in accordance with the UK Animals (Scientific Procedures) Act (1986). Experiments were carried out in accordance with ARRIVE guidelines. Experimental protocols were approved first by an Animal Welfare and Ethical Review Body at the University of Oxford, and then by a UK Government Home Office Project License. Wildtype male and female C57BL/6J mice (RRID: IMSR_JAX:000664) were used in this study. Mice were maintained under a 12-h light/12-h dark cycle to ensure entrainment. Ambient room temperature was maintained at 22 ± 2 °C and humidity at 50 ± 20%. At the relevant experimental time point, mice were euthanized either by cervical dislocation or by terminal dose of anesthetic (pentobarbital), which was then confirmed by cervical dislocation.

### Mouse organotypic cortical slice culture

Cerebrocortical organotypic slices cultures from C57BL/6J pups were generated as described previously, with slight modifications^73,74^. Pups at postnatal day 7 (P7) were rapidly decapitated and the brain was removed into freshly prepared ice-cold dissection media containing Earle’s balanced salt solution (EBSS, Gibco, Thermo Fisher Scientific) with calcium chloride and magnesium sulphate supplemented with 25.5 mM Hepes, 36.5 mM d-glucose, and 5 mM sodium hydroxide. The cerebral hemispheres were separated and the cortex along with subcortical structures was sectioned at 400 µm with a McIlwain Tissue Chopper. Slices were placed onto Millicell cell culture inserts (0.4 µm, 30 mm diameter) and were maintained at an interface between air and feeding media (containing 78.8% v/v Gibco Minimum Essential Media with GlutaMAX, 20% heat inactivated horse serum, 1% B27, 30 mM Hepes, 26 mM D-glucose, 5.8 mM sodium bicarbonate, 1 mM calcium chloride, 2 mM magnesium sulphate), and 1% pen/strep (containing 10,000 units of Penicillin-G, 10 mg Streptomycin and 25 µg Amphotericin B per ml) and incubated at 37 °C. After 3–5 days of recovery, slices were treated with either 5 µg/ml Tunicamycin or with vehicle alone (DMSO 1:1000) for 6 h, after which the treatment was replaced with fresh media. 18 h after treatment washout, slices were fixed with 4% PFA overnight and mounted onto glass slides using VectaShield mounting medium (Vector Labs).

## In utero electroporation

In utero electroporation (IUE) was performed using standard procedures^75^. Briefly, pregnant female C57BL/6 J mice were anaesthetized using isoflurane and their uterine horns were exposed by midline laparotomy. A mixture of sUPRa plasmid DNA (1.5 μg/μl) and 0.03% fast-green dye (Sigma Aldrich) was injected intraventricularly into each embryo through the uterine wall and amniotic sac with a glass micropipette (World Precision Instruments), pulled using a Flaming/Brown Micropipette Puller (Sutter Instrument Company). sUPRa plasmid DNA was prepared using the EndoFree Plasmid Kit (Qiagen, Hilden, Germany). The total volume injected per embryo was ~ 1 μl. Electroporation was performed by placing the anode of a Tweezertrode (Genetronics) over the dorsal telencephalon outside the uterine muscle. Five pulses (50 ms duration separated by 950 ms) at 42 V (at E14.5 and E15.5), 40 V (at E13.5), or 38 V (at E12.5) were delivered with a BTX ECM 830 pulse generator (Genetronics). The uterus was then lavaged with warmed, sterile Hartmann’s solution (Dechra Pharmaceuticals) and replaced into the abdomen. The abdominal muscle and skin incision were closed with Vicryl and Prolene sutures (Ethicon). Dams were allowed to recover in a clean cage and litter down naturally. The day of birth was taken as postnatal day (P) 0.

### Sleep deprivation and tissue collection

Eight 8 week-old male and female C57BL/6 J mice from 2 litters that had been in utero electroporated with sUPRa were used for sleep deprivation (SD) experiments. Animals were allowed 1 week to habituate to being singly housed and entrain to a 12-h light/dark cycle from 07:00 to 19:00 before experiments were carried out. SD was performed by experienced experimenters for 12 h starting at light onset, as described previously^76^. 12 h was selected as the duration of SD to allow enough time for sUPRa’s fluorescence signal to be induced, given the lag between transcriptional activation and translation. Mouse behavior and locomotion were constantly monitored, and upon signs of sleepiness the experimenter provided the animal with novel objects. Four mice underwent SD whilst four control mice were allowed to sleep undisturbed in a separate room. Following the 12-h SD period, mice were injected with a lethal dose of urethane and transcardially perfused with PBS followed by 4% PFA solution before tissue collection. Coronal Sects. (50 µm) were cut from each perfusion-fixed brain using a vibrating microtome (Leica VT1000S), and mounted onto glass microscope slides using VectaShield.

### Immunocytochemistry

For immunolabelling with anti-KDEL (ADI-SPA-827, Enzo Life Sciences, Clone 10C3), anti-RFP (5F8, Chromotek, lot #90228002AB) and anti-HA tag (Abcam, Cat # ab236632) antibodies, cells were first incubated in 10% goat serum, 0.3% TritonX-100 for 1 h at room temperature before being incubated with the primary antibodies diluted 1:500 in 5% goat serum in 0.15% TritonX-100 for 1 h at room temperature. After washing in 0.3% TritonX-100, the cells were then incubated with Alexa Fluor-conjugated IgG secondary antibodies (Life Technologies, Thermo Fisher Scientific) diluted in 5% goat serum in 0.15% TritonX-100 for 1 h at room temperature. After further washes, the cells were stained with DAPI (1:10,000 in PBS) for 5 min. Following a final wash, coverslips were mounted onto glass microscope slides using Prolong Diamond Antifade mountant (Invitrogen, Thermo Fisher Scientific).

### Imaging

NIH3T3 cells on coverslips were imaged using a 20 × objective (Olympus, UPlanFLN NA 0.5) on an epifluorescence microscope (Olympus BX40), equipped with a Hamamatsu ORCA-ER camera and HCImage Live software (GFP: 485/20–25 nm excitation filter, 506 nm dichroic, 524/24–25 nm emission filter; RFP: 540/40 nm excitation filter, 570 nm dichroic, 600/50 nm emission filter). Auto Hi-Lo and Contrast settings were turned off. Imaging parameters including exposure times were adjusted per fluorescent construct to avoid saturation, and were kept constant within each experiment. Confocal images of mouse brain cortical slices/sections were captured using a Zeiss LSM 880 confocal microscope, via a 20 × water-immersion objective (W Plan-Apochromat, NA 1.0), and controlled via ZEN software (Zeiss). sUPRa was excited at 561 nm using a diode-pumped solid-state laser, and at 488 nm using an argon laser. When exciting sUPRa at 561 nm, emitted fluorescence was collected by a photomultiplier tube (PMT) in the 570–670 nm range. When exciting sUPRa at 488 nm, emitted fluorescence was collected by a high sensitivity gallium arsenide phosphide PMT (GaAsP-PMT) in the 492–532 nm range. Z-stack images were taken from each slice/section at an interval of ~ 5 µm. Exposure times were carefully adjusted such that the fluorescence signals were not saturated, and kept constant across all slices/sections. Other settings such as pinhole aperture, optical zoom, laser intensity and dwell time were also kept constant. Tile scan images were taken from a segment of the cortex, spanning all cortical layers.

### Image analysis

NIH3T3 cells were identified from the UPR-independent fluorescence channel (mScarlet or anti-RFP), using a recently released Python package, Cellpose^77^. Segmentation “masks” generated by Cellpose were applied on the reporter fluorescence channel to obtain a sum fluorescence value for each cell. An averaged background fluorescence value, which was obtained for each image by averaging all of the pixels that were excluded from the cell masks, was subtracted from each cell. A threshold was applied such that cells with a background-subtracted fluorescence of zero or below were not included in subsequent analyses. Neurons expressing sUPRa in brain sections/slices were identified from the red mScarlet channel using the Cellpose package. The segmentation masks generated by Cellpose were applied on the mNeonGreen channel to obtain a sum fluorescence value for each cell. An averaged background value surrounding the cell masks was subtracted from each cell. There was a range of mScarlet expression levels, presumably reflecting variability in the number of plasmid copies that neurons inherited from their parent progenitor. To avoid issues with spectral bleed-through from the red into the green channel, neurons with extremely high red expression were excluded from subsequent analyses.

### Statistical analysis

All data were assessed for normality using the Shapiro Wilk test, following which the appropriate parametric or non-parametric statistical tests were applied. Statistical analyses were performed using the Scipy and Statsmodels packages in Python. Graphs were generated using Matplotlib and Seaborn packages in Python. Asterisks represent significant p-values following statistical tests (*p < 0.05, **p < 0.01 and ***p < 0.001).

### Supplementary Information

Supplementary Information.

## Data Availability

The sUPRa construct generated in this study is made available via Addgene (Addgene_223242; https://www.addgene.org/). Data reported in this paper are available from the corresponding author upon reasonable request.
