# Supplementary material for: sUPRa is a dual-color reporter for unbiased quantification of the unfolded protein response with cellular resolution
Source: Sci Rep. 2024 Jul 1;14:14990. doi: 10.1038/s41598-024-65611-2 (PMC11217371; doi:10.1038/s41598-024-65611-2)
Supplement: Supplementary file 1 — Supplementary Information. [file 41598_2024_65611_MOESM1_ESM.pdf]

Supplementary information

sUPRa is a dual-color reporter for unbiased quantification of the unfolded protein response with cellular resolution

Atreyi Chakrabarty[1], Sarah E. Newey[1], Maisha M. Promi[1], Belinda K. Agbetiameh[1], Daniella Munro[1], Paul J. N. Brodersen[1], Gemma Gothard[1], Kashif Mahfooz[1], Jose P. Mengual[2], Vladyslav V. Vyazovskiy[2], Colin J. Akerman[1]

[1]Department of Pharmacology, University of Oxford, Mansfield Road, University of Oxford, Oxford, United Kingdom, OX1 3QT

[2]Department of Physiology, Anatomy and Genetics, University of Oxford, Sherrington Building, Sherrington Road, Oxford, United Kingdom, OX1 3PT

Table S1: Genetic reporters of the mammalian UPR

Construct Name	UPR arm/target	Reporter	Comments	Reference
F-XBP1ΔDBD-venus	IRE1/ XBP1	The GFP variant Venus expresses in the cytosol upon unconventional splicing of XBP1 in response to IRE1 activation	Exploits XBP1 splicing-induced frameshift. Construct lacks the DNA binding domain (DBD) of XBP1 (prevents overexpression interfering in a dominant negative manner with endogenous UPR). Widely utilized in literature	Iwawaki et al., 2004
IRE1-reporter	IRE1/ XBP1	YFP expression upon XBP1 splicing, mainly localized in the nucleus	Utilises full length XBP1. In this case, overexpression reportedly does not disrupt endogenous IRE1-XBP1 signaling.	Walter et al., 2015
RXG	IRE1/ XBP1	Dual fluorescent reporter construct encoding RFP and GFP separated by a XBP1 splice site.	Constitutive CMV promoter drives RFP expression, enabling detection of both stressed and unstressed cells. Cells subject to ER stress result in XBP1 splicing causing a frameshift that places GFP in frame with RFP. UPR-active cells are both red and green.	Roy et al., 2017
XBP1-mNeonGreen-NLS<br><br>Addgene 115968, 115971	IRE1/ XBP1	Nuclear localized mNeonGreen expression upon XBP1 splicing	Utilises XBP1 minus DBD. Nuclear Localisation Signal (NLS) enables concentration of the fluorescent signal in the cell nucleus for automated live cell imaging. Available in lenti- and retroviral vector backbones	Nougarede et al 2018
CHOP::GFP<br><br>Addgene 21898	PERK/ CHOP	Transcriptional reporter incorporating CHOP promoter driving GFP	Contains 9.3Kb genomic region of the mouse CHOP gene upstream of exon 3 fused to GFP coding region.	Novoa et al., 2001
5’ATF4-GFP<br><br>Addgene 21852	PERK/ ATF4	Translational reporter that results in GFP expression on induction of ER stress.	Includes the ATF4 upstream open reading frames (uORFs) 1 and 2 which regulate ATF4 translation in response to ER stress.	Lu et al., 2004
PERK-reporter	PERK/ ATF4	Translational reporter that results in YFP expression on PERK activation	Includes ATF4 5’UTR region containing three uORFs cloned in frame with YFP. Low basal expression of YFP in unstressed cells.	Walter et al 2015
ATF4-mScarlet-NLS<br><br>Addgene 115969, 115970	PERK/ ATF4	Translational reporter that results in nuclear localized mScarlet expression on PERK activation	Exploits ATF4 ORF switch on PERK activation. NLS on mScarlet enables automated live cell imaging. Available in lenti- and retroviral vector backbones	Nougarede et al 2018

SPOTlight<br><br>Addgene 164819	PERK/ ATF4	Translational dual color fluorescent reporter of ER stress activation state.	Reporter differentially translates green (eGFP) or red (TdTomato) fluorescent proteins from a single ATF4 transcript based on ER stress activation state. Relies of differential usage of uORFs in the 5′untranslated region of ATF4. Generated in AAV backbone. TdTomato signal (reporting PERK activation) requires amplification with anti-RFP antibody.	Helseth et al., 2021
p5xATF6-GL3	ATF6	Luciferase reporter under the transcriptional control of a multimerized ATF6-binding site	Reporter contains 5x ATF6 binding sites in front of the c-fos minimal promoter.	Wang et al., 2000
Grp78 -169LUC/ -457LUC	IRE1 and ATF6 (-169LUC,-457LUC), and PERK (-457LUC)	Transcriptional reporters resulting in luciferase expression on activation of fragments of the BiP promoter.	-196LUC construct contains rat BiP promoter sequence -169 to -29bp upstream of transcriptional start site. Includes 3x ERSE elements that bind ATF6 and XBP1. -457LUC contains a promoter fragment from -457 to -29 and includes 3xERSE and CRE binding sites.	Luo and Lee, 2002; Luo et al., 2003, Mao et al., 2006
Grp78 3KbLacZ	IRE1 and ATF6 and PERK	Transcriptional reporter resulting in LacZ expression on activation of the BiP promoter	Reporter contains 3kb promoter region of the rat BiP gene driving LacZ. Useful BiP promoter deletion mutants also generated and used to generate transgenic mice.	Luo et al., 2003; Mao et al 2006
ERSE-tdTomato	IRE1 and ATF6	Transcriptional reporter resulting in TdTomato expression on activation of the ERSE element of the BiP promoter	Reporter contains -169bp to -29bp promoter fragment of rat BiP gene driving TdTomato. TdTomato is fused to ornithine decarboxylase sequence to target protein degradation.	Lajoie and Snapp 2011
BiP-mGFP<br><br>Addgene 62231	Misfolded protein burden	BiP protein mobility reporter using fluorescence recovery after photobleaching (FRAP)	Encodes hamster BiP protein fused to monomeric EGFP. Useful for studying BiP mobility and trafficking in live cells. BiP-mGFP protein decreases in mobility as it binds increasing amounts of misfolded proteins under ER stress. This can be detected nd quantified using confocal imaging and FRAP.	Lai et al., 2010<br><br>Lajoie and Snapp 2011

References:

Helseth AR, Hernandez-Martinez R, Hall VL, Oliver ML, Turner BD, Caffall ZF, Rittiner JE, Shipman MK, King CS, Gradinaru V, Gerfen C, Costa-Mattioli M, Calakos N. Cholinergic neurons constitutively engage the ISR for dopamine modulation and skill learning in mice. Science. 2021 Apr 23;372(6540):eabe1931. doi: 10.1126/science.abe1931. PMID: 33888613; PMCID: PMC8457366.

Iwawaki T, Akai R, Kohno K, Miura M. A transgenic mouse model for monitoring endoplasmic reticulum stress. Nat Med. 2004 Jan;10(1):98-102. doi: 10.1038/nm970. Epub 2003 Dec 14. PMID: 14702639.

Lai CW, Aronson DE, Snapp EL. BiP availability distinguishes states of homeostasis and stress in the endoplasmic reticulum of living cells. Mol Biol Cell. 2010 Jun 15;21(12):1909-21. doi: 10.1091/mbc.e09-12-1066. Epub 2010 Apr 21. PMID: 20410136; PMCID: PMC2883936.

Lajoie P, Snapp EL. Changes in BiP availability reveal hypersensitivity to acute endoplasmic reticulum stress in cells expressing mutant huntingtin. J Cell Sci. 2011 Oct 1;124(Pt 19):3332-43. doi: 10.1242/jcs.087510. Epub 2011 Sep 6. Erratum in: J Cell Sci. 2012 Feb 1;125(Pt 3):789. PMID: 21896647; PMCID: PMC3178454.

Lu PD, Harding HP, Ron D. Translation reinitiation at alternative open reading frames regulates gene expression in an integrated stress response. J Cell Biol. 2004 Oct 11;167(1):27-33. doi: 10.1083/jcb.200408003. PMID: 15479734; PMCID: PMC2172506.

Luo S, Baumeister P, Yang S, Abcouwer SF, Lee AS. Induction of Grp78/BiP by translational block: activation of the Grp78 promoter by ATF4 through and upstream ATF/CRE site independent of the endoplasmic reticulum stress elements. J Biol Chem. 2003 Sep 26;278(39):37375-85. doi: 10.1074/jbc.M303619200. Epub 2003 Jul 18. PMID: 12871976.

Luo S, Lee AS. Requirement of the p38 mitogen-activated protein kinase signalling pathway for the induction of the 78 kDa glucose-regulated protein/immunoglobulin heavy-chain binding protein by azetidine stress: activating transcription factor 6 as a target for stress-induced phosphorylation. Biochem J. 2002 Sep 15;366(Pt 3):787-95. doi: 10.1042/BJ20011802. PMID: 12076252; PMCID: PMC1222838.

Luo S, Mao C, Lee B, Lee AS. GRP78/BiP is required for cell proliferation and protecting the inner cell mass from apoptosis during early mouse embryonic development. Mol Cell Biol. 2006 Aug;26(15):5688-97. doi: 10.1128/MCB.00779-06. PMID: 16847323; PMCID: PMC1592753.

Mao C, Tai WC, Bai Y, Poizat C, Lee AS. In vivo regulation of Grp78/BiP transcription in the embryonic heart: role of the endoplasmic reticulum stress response element and GATA-4. J Biol Chem. 2006 Mar 31;281(13):8877-87. doi: 10.1074/jbc.M505784200. Epub 2006 Feb 1. PMID: 16452489.

Nougarède A, Tesnière C, Ylanko J, Rimokh R, Gillet G, Andrews DW. Improved IRE1 and PERK Pathway Sensors for Multiplex Endoplasmic Reticulum Stress Assay Reveal Stress Response to Nuclear Dyes Used for Image Segmentation. Assay Drug Dev Technol. 2018 Aug/Sep;16(6):350-360. doi: 10.1089/adt.2018.862. Epub 2018 Aug 8. PMID: 30088945.

Novoa I, Zeng H, Harding HP, Ron D. Feedback inhibition of the unfolded protein response by GADD34-mediated dephosphorylation of eIF2alpha. J Cell Biol. 2001 May 28;153(5):1011-22. doi: 10.1083/jcb.153.5.1011. PMID: 11381086; PMCID: PMC2174339.

Roy G, Zhang S, Li L, Higham E, Wu H, Marelli M, Bowen MA. Development of a fluorescent reporter system for monitoring ER stress in Chinese hamster ovary cells and its application for therapeutic protein production. PLoS One. 2017 Aug 23;12(8):e0183694. doi: 10.1371/journal.pone.0183694. PMID: 28832690; PMCID: PMC5568292.

Walter F, Schmid J, Düssmann H, Concannon CG, Prehn JH. Imaging of single cell responses to ER stress indicates that the relative dynamics of IRE1/XBP1 and PERK/ATF4 signalling rather than a switch between signalling branches determine cell survival. Cell Death Differ. 2015 Sep;22(9):1502-16. doi: 10.1038/cdd.2014.241. Epub 2015 Jan 30. PMID: 25633195; PMCID: PMC4532775.

Wang Y, Shen J, Arenzana N, Tirasophon W, Kaufman RJ, Prywes R. Activation of ATF6 and an ATF6 DNA binding site by the endoplasmic reticulum stress response. J Biol Chem. 2000 Sep 1;275(35):27013-20. doi: 10.1074/jbc.M003322200. PMID: 10856300.

Supplementary figures

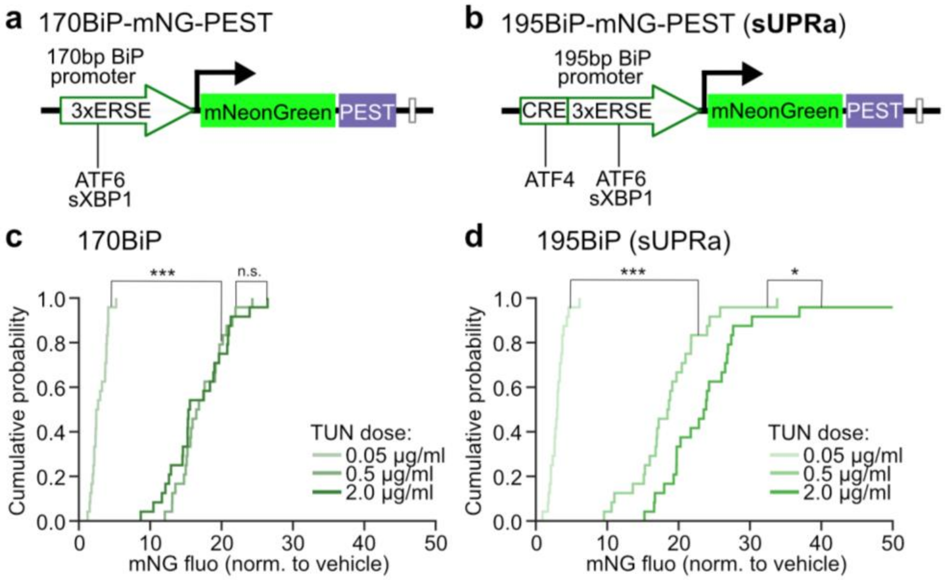

Figure S1: sUPRa is able to capture the difference in UPR activation between the highest tested doses of TUN, which is not captured by 170BiP

(A) Reporter construct driving expression of mNG-PEST using the 170bp (170BiP) region of the BiP promoter. **(B)** Reporter construct driving expression of mNG-PEST using the 195bp (195BiP) region of the BiP promoter, as used in sUPRa. NIH3T3 mouse fibroblasts were transfected with 170BiP or 195BiP and treated with either 1:1000 DMSO VEH or varying doses of TUN - an inducer of ER stress and the UPR - for 20 hours. **(C)** Cumulative distributions of mNG fluorescence in cells transfected with 170BiP-mNG-PEST and treated with 0.05, 0.5 or 2 µg/ml TUN, relative to VEH (n≈1250 cells from 24 FOVs from three experiments). 170BiP-mNG-PEST showed a significant increase in response between 0.05 µg/ml and 0.5 µg/ml TUN treatment (n=24 FOVs from three experiments; $p<0.001$, Kruskal Wallis test; $p<0.001$, Dunn's multiple comparisons test). However, there was no significant increase in response between 0.5 µg/ml and 2 µg/ml TUN ($p=0.09$ Dunn's multiple comparisons test). **(D)** Cumulative distributions of mNG fluorescence in cells transfected with 195BiP-mNG-PEST and treated with 0.05, 0.5 or 2 µg/ml TUN, relative to VEH (n≈1250 cells from 24 FOVs from three experiments). 195BiP-mNG-PEST showed a significant increase in response between 0.05 µg/ml and 0.5 µg/ml TUN treatment ($p<0.001$, Kruskal Wallis test; $p<0.001$, Dunn's multiple comparisons test) and between 0.5 µg/ml and 2 µg/ml TUN ($p=0.02$, Dunn's multiple comparisons test).

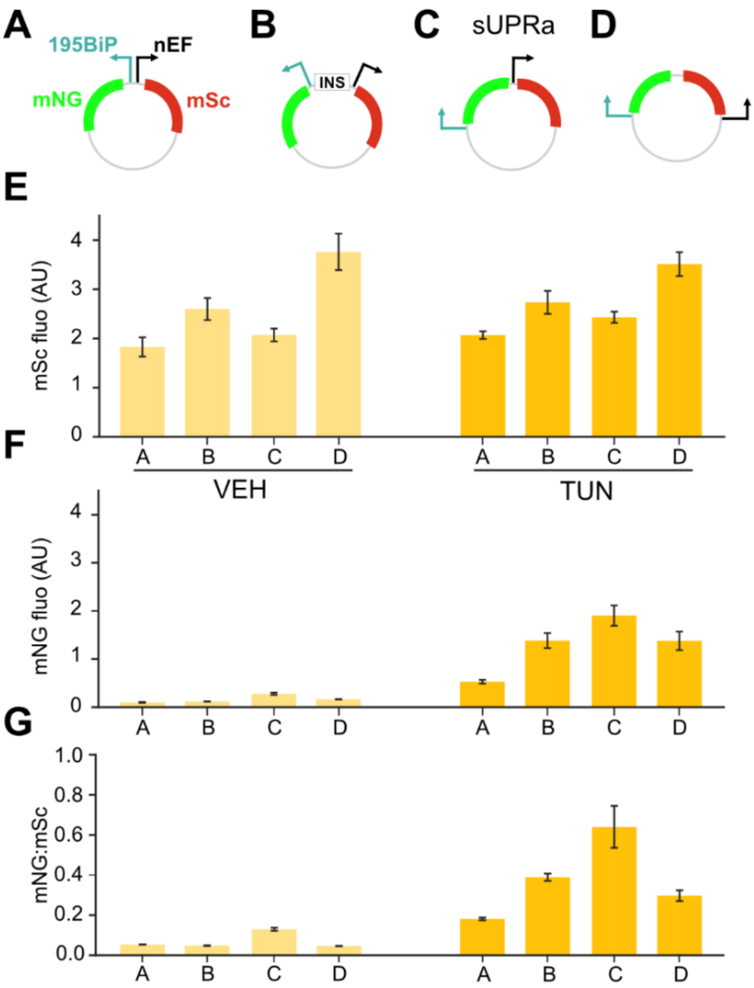

Figure S2: Optimizing the orientation of promoter sequences in sUPRa

sUPRa incorporates two expression cassettes: a fragment of the BiP promoter (195BiP) that drives the expression of mNeonGreen (mNG), and a constitutively active short EF1α promoter (nEF) that drives the expression of a second fluorophore, mScarlet (mSc), and enables the mNG signal to be normalized for cell-to-cell variability in reporter copy number. To optimize sUPRa, we tested different arrangements of the expression cassettes. **(A)** Schematic diagram of a construct using a back-to-back configuration. **(B)** A construct using a back-to-back configuration separated by an insulator (INS) sequence. **(C)** A construct with cassettes arranged sequentially, which was the final configuration selected for sUPRa. **(D)** A construct with cassettes facing one another. **(E)** NIH3T3 cells were transfected with one of the construct variants shown in '**A**' to '**D**' and treated with either VEH (left) or 0.5 µg/ml TUN (right) for 20 hours, then fixed and imaged. mSc fluorescence did not differ significantly between VEH and TUN treatments for all construct variants (n=8 FOVs from one experiment; $p>0.05$, *t* tests). **(F)** mNG fluorescence was significantly increased with TUN for all construct variants (n=8 FOVs from one experiment; $p<0.001$, *t* tests), and the mNG fluorescence intensity was greatest for variant '**C**' (sUPRa). **(G)** mNG:mSc fluorescence ratio was significantly increased with TUN for all construct variants (n=8 FOVs from one experiment; $p<0.001$, *t* tests), and was greatest for variant '**C**' (sUPRa).

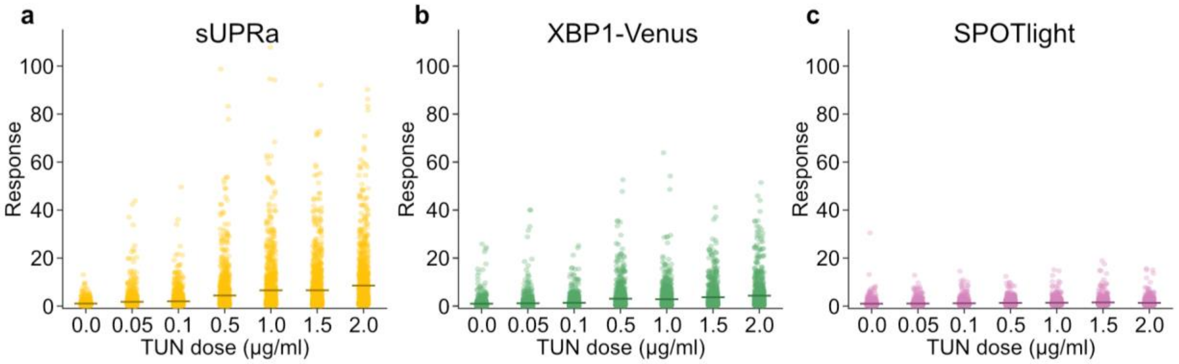

Figure S3: Dose-related responses to Tunicamycin at the individual cell level

(A) Cells from Figure 3C, expressing sUPRa and treated with a series of different TUN doses (0, 0.05, 0.1, 0.5, 1.0, 1.5 and 2 µg/ml) showed a dose-dependent response (n≈1200 cells per dose; $p<0.001$, Kruskal-Wallis test). **(B)** Cells from Figure 3C, expressing XBP1-Venus, showed a dose-dependent response to a series of TUN doses (n≈1000 cells per dose; $p<0.001$, Kruskal-Wallis test). **(C)** Cells from Figure 3C, expressing SPOTlight, showed a dose-dependent response to a series of TUN doses (n≈900 cells per dose; $p<0.001$, Kruskal-Wallis test).

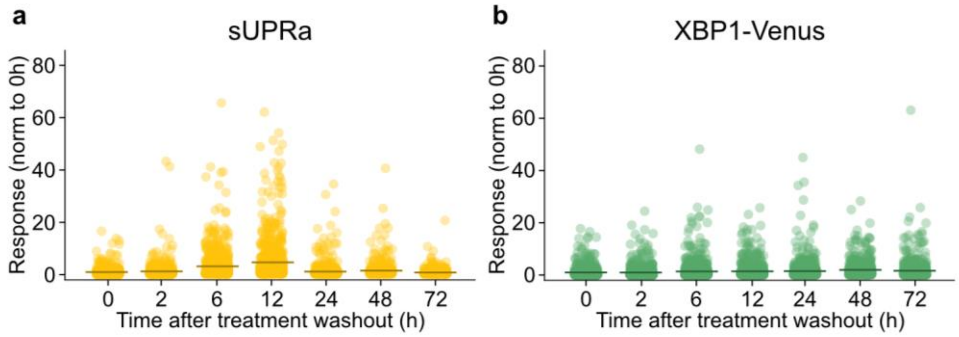

Figure S4: Response dynamics of sUPRa and XBP1-Venus at the individual cell level

(A) Cells from Figure 4C expressing sUPRa and treated with a 2-hour pulse of 2 µg/ml TUN, followed by treatment washout. The cells showed a gradual increase in the mNG:mSc ratio response, peaking at 12 hours and then recovering (n≈800 cells per dose; $p<0.001$, Kruskal-Wallis test). **(B)** Cells from Figure 4C expressing XBP1-Venus and treated with a 2-hour pulse of 2 µg/ml TUN followed by treatment washout. The cells showed a modest increase in Venus fluorescence response post-washout, which did not clearly recover by 72 hours (n≈1000 cells per dose; $p<0.001$, Kruskal-Wallis test).

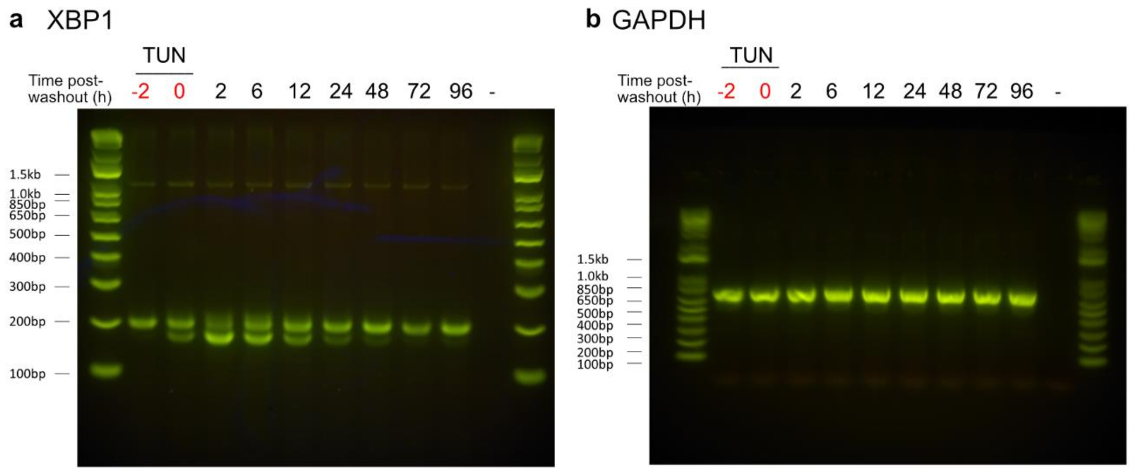

Figure S5: Endogenous XBP1 splicing in response to TUN treatment

(A) Uncropped image of agarose gels from Figure 4D, showing endogenous XBP1 transcript splicing in response to a 2-hour TUN treatment, before treatment washout and recovery after washout for 96-hours. The final lane shows the negative control with no template first strand. **(B)** Uncropped image of agarose gel from Figure 4D, showing GAPDH transcripts as a control RT-PCR for first strand amount and integrity, though bands were not used for normalisation. The 1kb DNA Plus ladder (Invitrogen) is shown with markers 100 bp - 1.5 kb.
